# Artificial intelligence for the measurement of vocal stereotypy

**DOI:** 10.1002/jeab.636

**Published:** 2020-11-03

**Authors:** Marie‐Michèle Dufour, Marc J. Lanovaz, Patrick Cardinal

**Affiliations:** ^1^ École de psychoéducation Université de Montréal; ^2^ Centre de recherche de l'Institut universitaire en santé mentale de Montréal; ^3^ Département de génie logiciel et des TI, École de technologie supérieure

**Keywords:** artificial intelligence, artificial neural network, autism, measurement, stereotypy

## Abstract

Both researchers and practitioners often rely on direct observation to measure and monitor behavior. When these behaviors are too complex or numerous to be measured in vivo, relying on direct observation using human observers increases the amount of resources required to conduct research and to monitor the effects of interventions in practice. To address this issue, we conducted a proof of concept examining whether artificial intelligence could measure vocal stereotypy in individuals with autism. More specifically, we used an artificial neural network with over 1,500 minutes of audio data from 8 different individuals to train and test models to measure vocal stereotypy. Our results showed that the artificial neural network performed adequately (i.e., session‐by‐session correlation near or above .80 with a human observer) in measuring engagement in vocal stereotypy for 6 of 8 participants. Additional research is needed to further improve the generalizability of the approach.

Whether experimental or applied, the science of behavior analysis targets a wide range of topics that aim to understand and to improve the functioning of human organisms (Skinner, 1951). A common thread central to this endeavor is the measurement of behavior. In most research involving human participants, researchers rely either on permanent products (e.g., responses automatically recorded from pressing on a computer screen) or on direct observation to examine the effects of independent variables on behavior. Practicing behavior analysts must also use these measures to monitor behavior when directly intervening with individuals in a professional setting (Behavior Analyst Certification Board, 2017).

The reliance on direct observation using human observers in many contexts raises an important issue related to available resources. For example, using continuous recording to monitor multiple or high frequency behavior often requires an independent observer who scores the behavior from a video recording. Furthermore, researchers need to include a second observer to increase the believability of the results by monitoring interobserver agreement (Mudford et al., 2009). If a researcher has 20 hr of video recordings to score for one participant, the human resources can easily add up to 30 to 50 hr of work. This time does not include the resources and time involved in hiring and training additional staff to conduct these tasks. With many participants, these additional resources can add up rapidly and limit the amount of research that can be done or the number of intervention sessions that can be afforded.

One potential solution to significantly reduce response effort associated with direct observation is to use artificial intelligence. Broadly, artificial intelligence is “the study of how to make computers do things at which, at the moment, people do better” (Rich & Knight, 1991, p. 3). As such, the measurement of behavior is a topic well suited to artificial intelligence as human observers are currently better than computers at monitoring most types of behavior (Goodwin et al., 2011). One promising tool in artificial intelligence is machine learning, which involves training models to detect signals or patterns in data (see Lanovaz et al., 2020, for behavior analytic introduction to the topic). That is, machine learning takes data as input to develop mathematical models that allows them to predict the value or categorization of novel data.

One type of machine learning algorithm is the artificial neural network. Simply put, an artificial neural network takes input data from the experimenter, which are then transformed by mathematical functions to produce a prediction (Goodfellow et al., 2016). Typically, artificial neural networks contain three types of layer: (1) the input layer, (2) the hidden layer, and (3) the output layer (see Fig. 1). The input layer receives features to train the model. The hidden neurons allow the model to learn more complex relationships between these features by transforming the data. Finally, the output layer provides the prediction of the model. Mathematically, the algorithm multiplies the input data by weights (which are initially set randomly) and transforms the product using an activation function to standardize the data. The result is then multiplied by a second series of weights and again transformed by an activation function. Next, the algorithm computes an error using a loss function, which compares the output values with the class labels (i.e., the true values). Finally, the algorithm retropropagates the gradient (derivative) of the error to update the weights. The retropropagation of the gradient of the error involves a variable called the learning rate, which determines how “fast” the model changes the weights. The retropropagation should produce updated weights that typically lead to more accurate predictions (less error) on the next pass. Each pass across the steps is called an epoch. The whole process is akin to shaping in behavior analysis where the model updates itself to provide increasingly more accurate responses following feedback.

**Figure 1:**
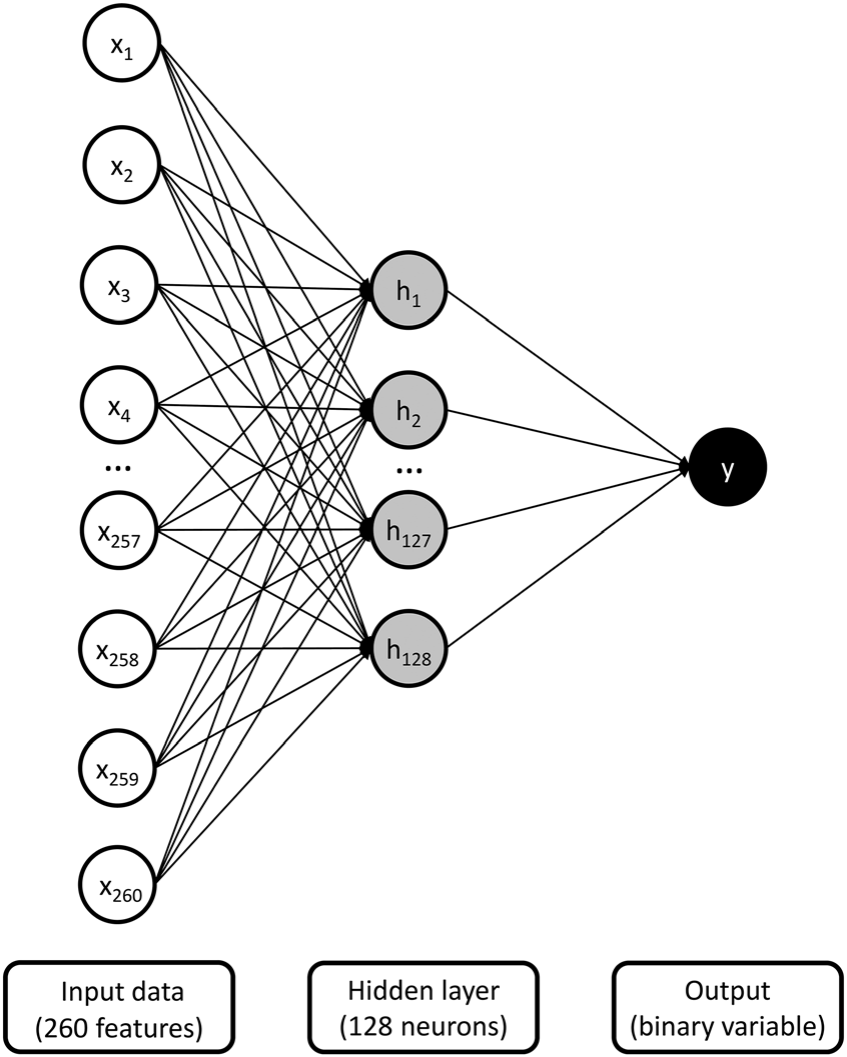
*Visual Representation of the Design of the Artificial Neural Network*

One challenge with artificial neural networks is that the training of models requires a large amount of data. Considering that individuals with developmental disability often engage in high rates of repetitive behavior, a starting point could be to apply these algorithms to this population. Individuals with developmental disability often engage in stereotypy, which is repetitive behavior characterized by movement invariance that is not maintained by social contingencies (Rapp & Vollmer, 2005). Researchers and practitioners further divide stereotypy into two types: motor and vocal stereotypy. Some researchers have already evaluated the use of machine learning algorithms to identify motor stereotypy in this population (Goodwin et al., 2011; Min & Tewfik, 2010; Rad & Furlanello, 2016; Westeyn et al., 2005).

In the first study on the automated detection of stereotypy, Westeyn et al. (2005) used accelerometers to monitor behaviors and then applied a hidden Markov model to classify the data. Their model was capable of automatically and accurately detecting 69% of hand flapping in one typically developing adult who was mimicking stereotypy. Following this study, other researchers applied different algorithms (k‐nearest neighbors and iterative subspace identification) to monitor body rocking and hand flapping in persons with autism with promising accuracy varying between 83% and 90% (Goodwin et al., 2011; Min & Tewfik, 2010). Finally, Rad and Furlanello (2016) applied artificial neural networks to detect motor stereotypy and found preliminary results that support the relevance of this approach.

The prior studies have all focused on motor stereotypy, but another form that should be targeted for reduction is vocal stereotypy (Rapp et al., 2013). A recent systematic review reported that 48% of individuals with developmental disability engage in at least one form of vocal stereotypy (Chebli et al., 2016). Examples of forms reported in the literature include monosyllable vocalizations, acontextual words or phrases, and acontextual laughing and grunting (DiGennaro Reed et al., 2012; Lanovaz et al., 2011; Rapp et al., 2013; Spencer & Alkhanji, 2018; Weston et al., 2018). Researchers have also evaluated the potential of machine learning for measuring vocal stereotypy (Min & Fetzner, 2018; 2019). In their first article, Min and Fetzner (2018) used a traditional machine learning algorithm to detect vocal stereotypy in four children with autism spectrum disorder (ASD) with an accuracy between 73% and 93%. In a second study, Min and Fetzner (2019) applied an artificial neural network to detect vocal stereotypy with an accuracy of 85%.

A serious limitation of both prior studies was that the researchers only measured whether stereotypy was absent or present in brief videoclips. If the procedures are to be useful to researchers and practitioners, we must develop models that can measure the duration of stereotypy (not only its presence or absence) during longer sessions. As a matter of fact, finding ways to automate the measurement of the duration of vocal stereotypy could not only decrease the amount of resources required for conducting research, but also facilitate the monitoring of the behavior in applied settings. A second limitation is that the researchers only extracted videos that did not contain treatment (akin to baseline). Researchers and practitioners are likely to measure vocal stereotypy in baseline and treatment sessions, which is why examining the models under both these conditions is important. One common treatment for vocal stereotypy is providing access to noncontingent music (i.e., continuous preferred music). Researchers have repeatedly shown that this treatment is effective at reducing vocal stereotypy in children with ASD (Gibbs et al., 2018; Lanovaz et al., 2011; Saylor et al., 2012). Given the potential challenges of identifying vocal stereotypy during this treatment condition, the inclusion of such sessions when testing models appears essential.

Thus, the purpose of our study was to determine whether we could train a machine learning algorithm to measure the duration of vocal stereotypy using audio data extracted from video recordings of both baseline and treatment sessions. More specifically, our study examined whether models derived from an artificial neural network could produce session‐by‐session correlations at or above .80 when compared to the values measured by a human observer. We set the benchmark at .80 because (a) this correlation score is considered strong to very strong (Schober et al., 2018), and (b) prior research has found similar or higher correlations when examining the validity of discontinuous measurements methods (Leblanc et al., 2020). Given the purpose of our study, our research questions were:

1. Can an artificial neural network produce a session‐by‐session correlation of .80 or better with human observers when measuring the duration of vocal stereotypy?
2. What type of data analysis (i.e., within‐participant, between‐participant, or hybrid approach) produces the best measures of vocal stereotypy?

## Method

### Participants and Settings

To train and test our models, we measured vocal stereotypy in eight children with ASD who had previously participated in a study on the use of a mobile app to reduce engagement in stereotypy (Préfontaine et al., 2019). The mobile app involved the delivery of noncontingent music for children who engaged mainly in vocal stereotypy. Two participants, Dave and Emile, also received differential reinforcement as an intervention during some of the sessions. An independent multidisciplinary team had provided a diagnosis of ASD to each child prior to their participation in the study. All participants received their schooling or their primary care in English or French. Table 1 presents the age, gender, and a description of the vocal stereotypy for each participant. Our university research ethics board approved both the original and the current study.

**Table 1:** *Participant Characteristics*

Participants	Age	Gender	Description of vocal stereotypy
Emile	7	M	Grunting and unintelligible vocalizations
Matt	5	M	Monosyllable sounds and repetitive singing
Dave	6	M	Humming and unintelligible vocalizations
Billy‐Peter	8	M	Monosyllable sounds and acontextual giggling
Owen	7	M	Phrase or word repetitions
Dan	11	M	Phrase or word repetitions
Alia	10	F	Humming and unintelligible vocalizations
Nate	6	M	Phrase or word repetitions

The sessions occurred in each child's home during regularly planned activities, which affected the quality of audio recordings. Therefore, we excluded sessions (a) where siblings could be heard in the background because the first author was unable to determine which sounds were emitted by whom when relying on the audio recordings, and (b) in which ambient noise or sounds impeded the measurement of vocal stereotypy by the first author. Table 2 presents the number of sessions and the time in seconds of recordings for each participant that we used as part of the current study.

**Table 2:** *Number of Sessions and Duration of Dataset Per Participant*

Participants	Number of Sessions	Total Time (s)	With Music (s)	Without Music (s)
Emile	38	27,448	719	26,729
Matt	6	4,015	2,044	1,971
Dave	30	20,756	3,685	17,071
Billy‐Peter	10	6,909	2,729	4,180
Owen	25	17,461	4,357	13,104
Dan	11	7,533	4,750	2,783
Alia	10	7,091	2,783	4,308
Nate	12	8,351	4,820	3,531
**Total**	142	99,564	25,887	73,677

### Extraction of Audio Recordings and Features

The first author extracted the audio from standard definition video recordings in .mpeg format using VLC®, an open source video software. The program extracted the audio to a .wav format with a sampling rate of 22,050 Hz, one audio channel and a bit rate of 16 Kbits/s. Both the human observers used this .wav file to measure vocal stereotypy. Artificial neural networks cannot analyze .wav files because the amount of information encoded is too large for processing with a typical computer. To address this issue, we used the package python_speech_features for Python to extract the Mel Filterbank Cepstrum Coefficient (MFCC) from the audio files. The MFCC allows the extraction of a set of 26 audio features that have been widely used in machine learning, specifically in speech recognition (Chia et al., 2012; Kumar et al., 2011). These 26 features provide a description of the sound during short time windows. Our algorithm sampled the sound every 0.1 s for a time window of the same duration. Therefore, each second of recording was represented by 260 features (10 timesteps multiplied by 26 features).

### Artificial Neural Networks

As part of the current study, we selected an artificial neural network as our machine learning algorithm. Our raw data and Python code are freely available on the Open Science Framework (see https://osf.io/e4vbs/). Figure 1 depicts our artificial neural network. The input involved 260 features per second of recording (see extraction of audio recordings and features section). Based on broad recommendations by Heaton (2015) and on the computation power available to us, our neural network contained a single hidden layer with 128 neurons. Our model involved a single binary output value for each second: vocal stereotypy present (value = 1) or vocal stereotypy absent (value = 0). For our analysis, we used the Adam optimizer to set our learning rate. The algorithm trained our models until the kappa metric (see below) had not improved for 10 consecutive epochs (i.e., loops) on the validation set. Given that our audio recordings contained more seconds containing silence than vocal stereotypy, we also applied a correction to the error to balance them out. To promote generalization to untrained exemplars, our algorithm also applied dropout regularization, which randomly left out 20% of the data in each layer when making predictions.

### Data Collection and Interobserver Agreement

The first author manually coded each audio recording on a second‐by‐second basis using Audacity®, an open source audio software. We defined vocal stereotypy as acontextual or unintelligible sounds or words produced by the vocal apparatus of the child. If vocal stereotypy was present even for a fraction of a second, the first author coded the behavior as occurring during the second (as in partial interval recording with 1‐s intervals). Otherwise, she scored the behavior as not occurring during the second. A second observer measured vocal stereotypy on 42% of the recordings. The mean second‐by‐second interobserver agreement was 97% (range: 93%‐99%) and the mean kappa interobserver agreement was .87 (range: .81‐.94).

### Procedures

#### Between‐Participant Analysis

Our first analyses aimed to determine whether our models could predict the duration of vocal stereotypy for children whose data were not used during training. If the model produced adequate predictions, behavior analysts could develop models that could be applied to any child who engages in vocal stereotypy. To conduct the analysis, we used a variation of the leave one out cross‐validation methodology (Wong, 2015). Our code divided our participants into three sets: the training set (six participants), the validation set (one participant), and the test set (one participant). Our algorithm used the training set to train and update the model, and the validation set to determine when to stop the training and to select the model that produced the highest kappa value. The test set assessed generalization, as it was not used during training or to select the most accurate model. The program repeated our analyses eight times so that each participant was in the test set once, and in the validation set once.

For each model, we measured accuracy, the kappa statistic and a correlation on the test (generalization) set. To measure accuracy, the code divided the number of seconds on which the prediction of our model and the observation of the first author agreed by the total number of seconds in the dataset. Accuracy can be easily skewed by unbalanced datasets by which a model is better at predicting the absence than the presence of vocal stereotypy. Therefore, we also measured agreement using the kappa statistic as it provides control over agreements that are the result of chance and balances the values of both possible outcomes. Kappa and accuracy only provide a within‐session measure of agreement. Behavior analysts typically consider session‐by‐session patterns when analyzing single‐case data in graphs. To address this issue, we added a measure comparing session‐by‐session values. Specifically, our program measured correlations on a session‐by‐session basis between the percentage of vocal stereotypy computed by the model and the percentage of stereotypy observed by the first author. To examine the potential effects of music on measurement, the previous analyses were repeated twice: once with a dataset including all sessions and a second time with a dataset excluding sessions with music.^1^

#### Within‐Participant Analysis

Our second series of analyses involved examining whether we could produce better results using within‐participant predictions. If the model produced adequate predictions, behavior analysts could score the first few sessions of a child for vocal stereotypy and then use the model to predict vocal stereotypy in subsequent sessions. In this case, we conducted the analysis for each participant individually. The test set contained a single session for the participant whereas the remaining sessions were divided between the training set (83% of the remaining sessions) and the validation set (17% of the remaining sessions). The code repeated the analysis once per session for each participant. Apart from the composition of the test, training, and validation sets, the procedures and analyses remained the same as for the between‐participant analyses. Moreover, we did not test for the effects of music because the amount of data would have been insufficient for many participants. As our analysis produced multiple values for each participant, the means across sessions are reported.

#### Hybrid Analysis

Our third series of analyses involved combining within‐ and between‐participant data. As in the within‐participant analysis, the test set contained a single session for the participant, whereas the remaining sessions were divided between the training set (67% of the remaining sessions) and the validation set (33%^2^ of the remaining sessions). However, we also added between‐participant data in such a way that the training set contained 50% of within‐participant data and 50% of data from other participants, which increased the number of samples. Because the between‐participant data had a lot more samples, the algorithm picked the samples randomly to match the number from the within‐participant component.

## Results

As shown in Table 2, there were between six and 35 sessions per participant, for a total of 142 sessions from eight participants. The total duration of the 142 sessions was 99,564 s. The total duration of sessions with music was 25,887 s, whereas the duration of sessions without music was 73,677 s (see Table 2). We used the previous data to develop our machine learning models using between‐participant, within‐participant, and hybrid analyses.

**Figure 2:**
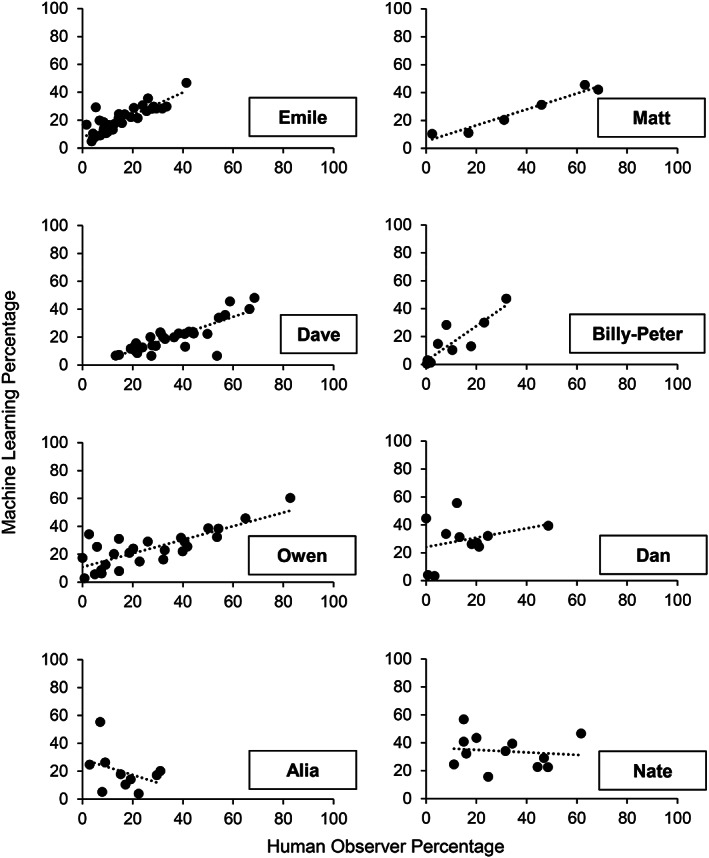
*Between‐Participant Analyses: Correlation Between the Percentages Measured by the Machine Learning Algorithm and Those Measured by the Human Observer Across All Sessions for Each Participant*

**Figure 3:**
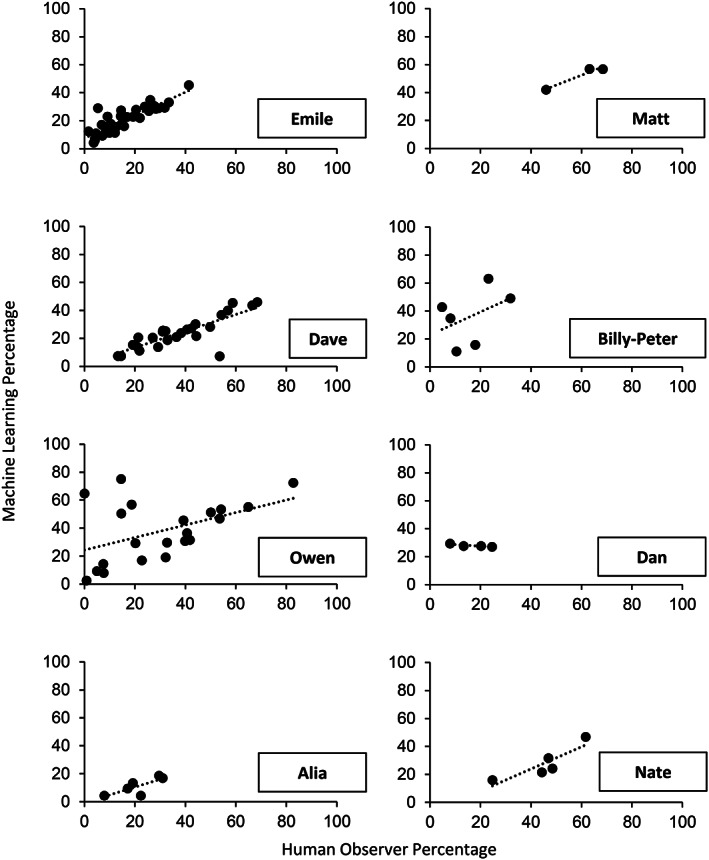
*Between‐Participant Analyses: Correlation Between the Percentages Measured by the Machine Learning Algorithm and Those Measured by the Human Observer Across Sessions Without Music Only for Each Participant*

The left side of Table 3 and Figure 2 present the results of the between‐participant analyses. Five of the eight participants had kappa statistics above or close to 0.5, indicating moderate to substantial agreement between the human observer and the computer model. For these five participants, the session‐by‐session correlation between the human observer and the computer model remained above .80, which indicates a strong to very strong correlation (see Fig. 2). Two participants (i.e., Alia and Nate) had negative correlations, which indicates that models were more likely to produce an inverse pattern when compared to the true values. Therefore, we repeated the analysis on sessions without music only to determine whether the background music was misleading the algorithms (see right side of Table 3 and Fig. 3). For Alia and Nate, all measures improved. However, the removal of music sessions considerably worsened the correlations for three participants (i.e., Billy‐Peter, Owen, and Dan).

**Table 3:** *Between‐Participant Analyses: Accuracy, Kappa, and Correlation for All Sessions and Those Without Music Sessions*

	All Sessions			Sessions Without Music Only		
Participants	Accuracy	Kappa	Correlation	Accuracy	Kappa	Correlation
Emile	0.90	0.66	0.86	0.90	0.67	0.87
Matt	0.78	0.49	0.97	0.77	0.54	0.97
Dave	0.79	0.50	0.82	0.81	0.57	0.81
Billy‐Peter	0.89	0.50	0.88	0.73	0.33	0.42
Owen	0.83	0.52	0.80	0.78	0.50	0.47
Dan	0.75	0.29	0.30	0.77	0.34	‐0.90
Alia	0.79	0.30	‐0.37	0.87	0.52	0.78
Nate	0.71	0.33	‐0.12	0.79	0.57	0.88

**Figure 4:**
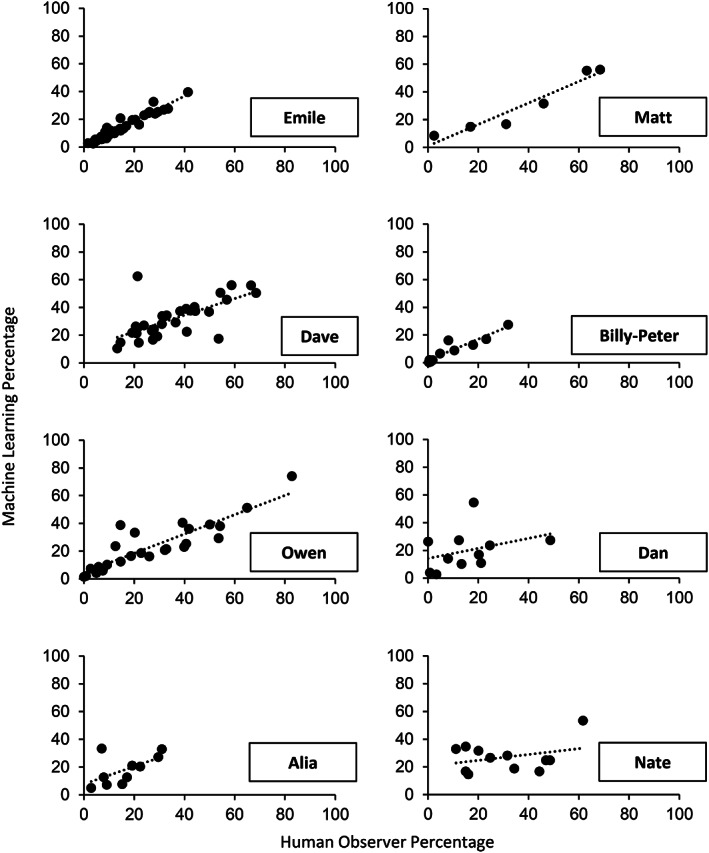
*Within‐Participant Analyses: Correlation Between the Percentages Measured by the Machine Learning Algorithm and Those Measured by the Human Observer Across Sessions for Each Participant*

Table 4 and Figure 4 present the results of the within‐participant analyses. Rather than using the data from the other participants to train the models (as in our between‐participant analyses), the within‐participant analyses consisted of training the models with the participant's own data. This manipulation involved a tradeoff: It reduced the amount of data available in the training set for each participant, but it also made the training set more like the vocal stereotypy that we were trying to measure. The results show that the kappa statistics were higher in the within‐participant analysis than the between‐participant analysis for four participants. In contrast, the correlations improved for six of eight participants. A further examination of these data indicates that this result may be misleading. This improvement involved the three participants who had the lowest correlations in the between‐participant analyses. As such, fewer participants achieved the .80 correlation criterion in the within‐participant analyses (i.e., four) than in the between‐participant analyses (i.e., five).

**Table 4:** *Within‐Participant Analyses: Accuracy, Kappa, and Correlation for Each Participant*

Participants	Accuracy	Kappa	Correlation
Emile	0.94	0.75	0.97
Matt	0.80	0.43	0.96
Dave	0.83	0.60	0.66
Billy‐Peter	0.91	0.25	0.93
Owen	0.86	0.40	0.88
Dan	0.79	0.23	0.34
Alia	0.91	0.67	0.58
Nate	0.74	0.34	0.33

**Figure 5:**
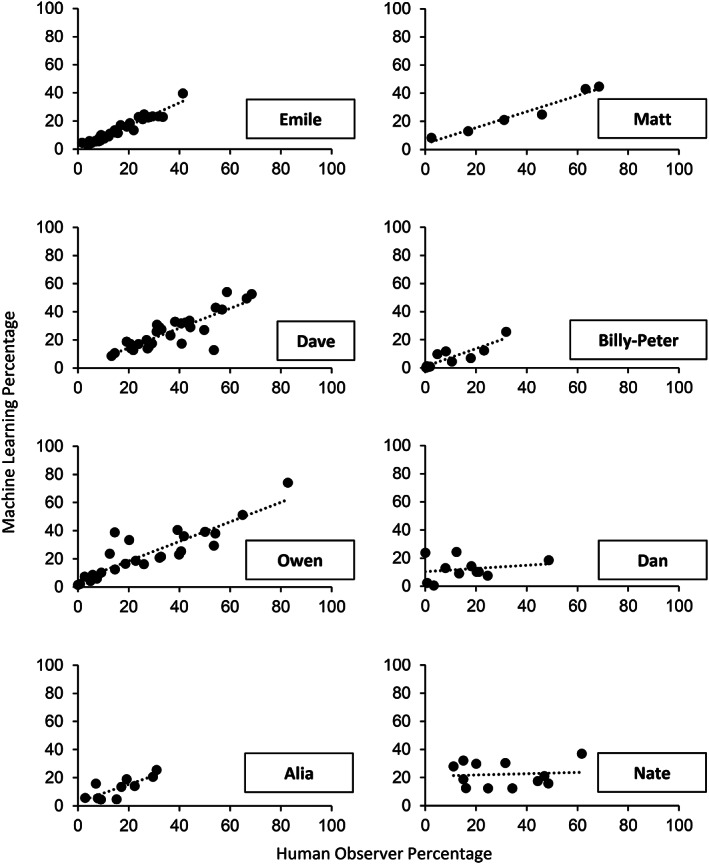
*Hybrid Analyses: Correlation Between the Percentages Measured by the Machine Learning Algorithm and Those Measured by the Human Observer Across Sessions for Each Participant*

As discussed previously, one issue with within‐participant analyses is that the training sets were smaller than in the between‐participant analyses (i.e., anywhere between 4% and 27% of the total number of samples in the dataset). To address this concern, we further conducted an analysis using a hybrid method combining the within‐ and between‐participant analyses. Table 5 and Figure 5 show the results of the hybrid analyses. Adding between‐participant data to the within‐participant models increased correlations to near or above .80 for two more participants (i.e., Dave and Alia), which led to the models adequately predicting session‐by‐session patterns for six of eight participants.

**Table 5:** *Hybrid Approach: Accuracy, Kappa, and Correlation for Each Participant*

Participants	Accuracy	Kappa	Correlation
Emile	0.95	0.74	0.97
Matt	0.78	0.41	0.98
Dave	0.83	0.57	0.84
Billy‐Peter	0.91	0.23	0.87
Owen	0.85	0.45	0.88
Dan	0.83	0.24	0.20
Alia	0.92	0.60	0.79
Nate	0.73	0.31	0.08

**Figure 6:**
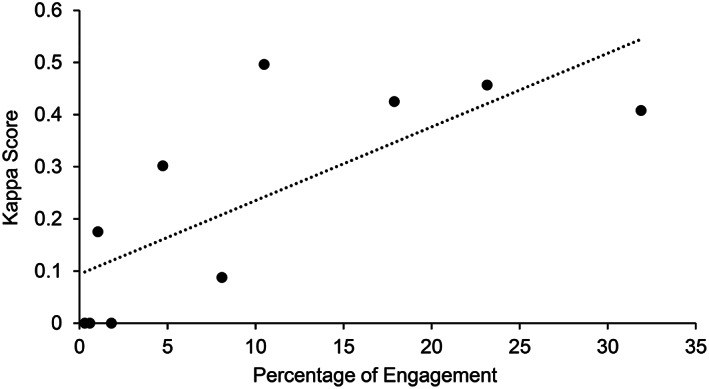
*Correlation Between the Kappa Scores and the Percentage of Engagement in Each Session for Billy‐Peter*

An unexpected observation from the previous analyses was that the kappa scores did not necessarily increase when correlations increased. Kappa represents within‐session patterns of responding, whereas correlations capture between‐session patterns (e.g., immediacy, level, trend). One potential explanation is that errors in measurement in sessions with low levels of stereotypy may deflate the mean kappa scores. As an example, assume that a human observer measured a behavior for 0.3% of a session whereas the model did not detect the behavior (i.e., 0%). Despite the absolute difference being only 0.3%, the kappa score would be 0 for this session. To examine this hypothesis, we measured the correlation between the kappa score and the percentage of engagement on a session‐by‐session basis. Figure 6 shows an example of this correlation for Billy‐Peter.^3^ Our analyses found a positive correlation between kappa and percentage of engagement for all participants, indicating that sessions with low levels of stereotypy skewed the estimation of the kappa scores towards lower values (as the computation of the reported kappa scores involved the mean of all sessions).

## Discussion

Our proof of concept produced session‐by‐session correlations near or above .80 for six of eight participants when using a hybrid approach, which generally produced the best outcomes. The hybrid approach may have performed best because it took advantage of each participant's individual responding while augmenting the dataset with samples from other participants. Interestingly, the removal of music during the between‐participant analyses significantly improved the measures for two further participants while worsening the predictions for three others. This worsening of the results may be explained by the removal of the data from the music sessions. The algorithm trained and tested the models on less data in the sessions without music only, which could explain the reduction for some participants. Nonetheless, the results are encouraging, as the high correlations observed in the hybrid analyses included both baseline and treatment sessions.

As noted in the results, the analyses often produced better estimations of between‐session patterns than within‐session patterns, which may be partly caused by the difficulty in estimating low levels of behavior. This result is consistent with prior research by Leblanc et al. (2020) who found that discontinuous methods of measurement produced less accurate estimates when challenging behavior occurred less frequently. Another potential explanation is that machine learning may produce systematic minor errors at the within‐session level that have a limited effect at the between‐session level. This type of systematic error is not unheard of in behavior analysis. One notable example is the use of discontinuous recording methods. Although discontinuous methods may produce considerably different within‐session patterns, between‐session patterns are similar enough to make these tools useful in practice (LeBlanc et al., 2020; Meany‐Daboul et al., 2007; Rapp et al., 2008; Schmidt et al., 2013). Similarly, our machine learning models preserved important between‐session features used for the analysis of single‐case designs, such as level, trend, and immediacy, while producing less consistent within‐session patterns.

To our knowledge, this is the first study to use artificial intelligence algorithms to measure the duration of vocal stereotypy during sessions. Our results replicate and extend prior studies that have used machine learning to measure motor and vocal forms of stereotypy (Goodwin et al., 2011; Min & Tewfik, 2010; Rad & Furlanello, 2016; Westeyn et al., 2005). We also extend research on artificial intelligence, as we studied how we can program computers to perform a task at which humans are currently better. Notably, some of our models produced session‐by‐session correlations that rivaled those produced by discontinuous measurement methods (Leblanc et al., 2020). Despite the promising nature of our results, we consider our study as an experimental proof of concept because the session‐by‐session correlations remained inadequate for two participants. As researchers working in a university (nonclinical) setting, we simply did not have access to sufficient data to further improve the performance of our models.

Our artificial neural network trained the models on audio data extracted from video recordings. Hence, the distance between the microphone and the child varied within and across sessions, rendering the analysis by the algorithms more challenging. In the future, we recommend that researchers use a wireless microphone positioned on the child's shirt collar, which should considerably improve measurements by increasing the power of the signal produced by the child's vocal apparatus. Moreover, this change would also facilitate the discrimination between the child's sounds and those of other individuals in the environment. If researchers continue improving the current models, the use of artificial intelligence may produce significant changes in research and practice such as the reduction of costs and the automation of certain repetitive tasks. With additional research, we can imagine the development of systems that could automatically measure target vocal behavior within research, educational and clinical contexts, freeing up time for researchers and practitioners to focus on other important activities. The utility of these models could move beyond single‐case designs. Researchers could also use automated measures with large randomized samples.

There are two additional limitations that should be noted. First, we used a single method and set of hyperparameters to extract the audio and train our models because we lacked the computing power to conduct multiple comparison analyses. Evaluating the effects of the extraction method and hyperparameters on algorithm performance with more powerful computers (or supercomputers) would be relevant in the future. Second, we did not examine and compare patterns on single‐case graphs. Instead, we used a correlation measure that is similar to a recent study examining correspondence between continuous and discontinuous measurements (see Leblanc et al., 2020). Given that we had to remove sessions due to low‐quality recordings, the sessions were not necessarily consecutive, preventing a thorough single‐case graph analysis. That said, the ultimate litmus test for our approach will be whether functional relations remain observable on single‐case graphs when applying these algorithms in research and applied settings.

## Endnotes

^1^
Emile wore headphones during most of the noncontingent music sessions. For analyses purposes, these sessions were categorized as having no music because the audio recordings contained no background music.

^2^
We could add more sessions in the validation set because our training set was larger due to between‐participant data.

^3^
We chose Billy‐Peter as an example because he showed the largest discrepancy between kappa and correlation scores.
